# COI DNA Barcoding of Six Schizothoracine Fishes from the Tarim River Basin, Xinjiang, China: Implications for Species Delimitation and Phylogenetic Relationships

**DOI:** 10.3390/biology15120894

**Published:** 2026-06-06

**Authors:** Dandan Zhang, Pengtao Liu, Xiaoming Lu, Huimin Hao, He Sun, Zhulan Nie, Shengjie Ren

**Affiliations:** 1 College of Life Science and Technology, Tarim University, Alar 843300, China; 18449600718@163.com (D.Z.); liupengtao@taru.edu.cn (P.L.); 13755247590@163.com (X.L.); 18835739177@163.com (H.H.); 120060006@taru.edu.cn (Z.N.); 2 State Key Laboratory Incubation Base for Conservation and Utilization of Bio-Resources in the Tarim Basin, Alar 843300, China; 3 Haida Feed Co., Ltd., Chongqing 402460, China; sh1014943501@163.com

**Keywords:** barcode gap, genetic divergence, haplotype diversity, mitochondrial lineages, species delimitation

## Abstract

To test the performance of *COI*-based DNA barcoding in species delimitation, we amplified and sequenced the mitochondrial *COI* gene from six Schizothoracine fishes endemic to Xinjiang, China. We then characterized the *COI* gene dataset, quantified genetic divergence, and inferred phylogenetic relationships using distance-based approaches. Morphological examination supported clear phenotypic differentiation among taxa. In particular, *Diptychus maculatus* can be readily distinguished from the densely scaled *Schizothorax* species by having a single pair of barbels and relatively sparse scales. The *COI* gene sequences showed an evident AT bias (55.1%), consistent with patterns reported for teleost mitochondrial genomes. Across all samples, 11 haplotypes were identified. Pairwise comparisons indicated a maximum interspecific divergence of 15.296%, whereas the smallest interspecific distance (0.262%) occurred between *Schizothorax barbatus* and *Schizothorax irregularis*. Species-delimitation analyses and phylogenetic reconstruction supported *D. maculatus* and *A. laticeps* as distinct mitochondrial lineages, whereas *S. biddulphi*, *S. eurystomus*, *S. irregularis*, and *S. barbatus* were not fully resolved by *COI* gene. The shared haplotypes and low genetic distances among the four *Schizothorax* species may reflect recent divergence, incomplete lineage sorting, or historical gene flow. Overall, *COI* gene barcoding is effective for distinguishing the major lineages in this dataset, especially *D. maculatus* and *A. laticeps*.

## Simple Summary

Correctly identifying fish species is essential for protecting freshwater biodiversity, but it can be difficult when closely related fishes look similar. This study aims to test whether physical characteristics and a short genetic marker can help distinguish six related fish species from the Tarim River Basin in Xinjiang. We examined 124 fish and found that two species, *Diptychus maculatus* and *Aspiorhynchus laticeps*, could be clearly separated by both appearance and genetic evidence. However, four *Schizothorax* species shared very similar genetic patterns, suggesting that this single genetic marker cannot fully separate them. These results show that genetic testing is useful for fish identification, but it should be combined with careful examination of body features when species are closely related. The study provides practical reference information for monitoring native fishes, protecting threatened species, and supporting better management of freshwater resources in Xinjiang.

## 1. Introduction

Schizothoracine fishes (Cyprinidae: Schizothoracinae) represent one of the most characteristic fish groups of the Qinghai–Tibet Plateau, with distributions extending into neighboring high-altitude river networks. A diagnostic feature of the genus is the abdominal slit positioned between two rows of enlarged anal scales, which has long been considered an adaptation to plateau environments [1]. In arid northwestern China, Xinjiang constitutes an important refugium for inland freshwater fauna and supports six widely recognized Schizothoracine taxa: *Schizothorax biddulphi* Günther, 1876 [2], *Aspiorhynchus laticeps* Day, 1877 [3], *Diptychus maculatus* Steindachner, 1866 [4], *Schizothorax irregularis* Day, 1877 [5], *Schizothorax barbatus* McClelland & Griffith, 1842 [6], and *Schizothorax eurystomus* Kessler, 1872 [7]. Although these fish underpin local fisheries, they are also tightly linked to the ecological functioning of plateau river systems and can reflect changes in regional hydrological conditions. In the Tarim Basin, long-term changes in fish fauna have been associated with both biological invasion and human disturbance. Chen et al. reported that 63 fish species have been recorded in the basin, including 19 native species and 44 alien species, and suggested that alien fishes may contribute to the decline of native fishes through predation and competition [8]. Human-mediated disturbances, particularly habitat degradation and overexploitation, together with climate-related hydrological alterations, have contributed to substantial population declines in recent years [9]. *Aspiorhynchus laticeps* has been treated as a threatened fish, and overfishing has been recognized as a potential cause of population decline and a serious complication for its conservation. Hydrological alteration has also been quantitatively documented in the Tarim River: Xue et al. showed that reservoir irrigation and channel irrigation substantially altered the river’s monthly flow regime, with average monthly flow decreasing in most months, especially in March, June, and August [10]. In addition, glacier retreat driven by climate warming has been documented in the Tarim River Basin, where glaciers play a significant role in the regional water-resource system; long-term glacier wastage has been shown to affect water availability in this arid inland basin [11]. Their slow life histories—especially low fecundity and late maturation—further limit recovery potential once populations are reduced [12]. Conservation concerns are reflected in national protection listings: *D. maculatus* was added as a Class II protected species in 2021 following habitat deterioration, population collapse, and body-size reduction [13], whereas *A. laticeps* has been protected as a Class I species since 1988, reflecting its high conservation priority and scientific importance [14].

However, the taxonomy and genetic diversity of Schizothoracine fishes in Xinjiang remain insufficiently resolved. In particular, several nominal *Schizothorax* species from the Tarim River Basin are morphologically similar and are mainly distinguished by subtle characters such as lip structure, barbel number, and keratinization of the lower jaw. These characters may vary with ontogenetic stage, habitat conditions, or phenotypic plasticity, making species identification difficult when relying only on external morphology. More importantly, it remains unclear whether currently recognized *Schizothorax* species in this region represent independently evolving genetic lineages or whether some nominal species reflect recently diverged populations, ecotypes, incomplete lineage sorting, or introgressed lineages. Recent species-delimitation studies have emphasized that morphological and mitochondrial evidence may become discordant when recent divergence, incomplete lineage sorting, or gene flow is present [15,16]. Therefore, clarifying the extent of genetic divergence and haplotype sharing among Tarim Basin *Schizothorax* species is necessary for both taxonomy and conservation.

Morphology remains the foundation of fish taxonomy, yet reliable identification becomes difficult when species show extensive phenotypic plasticity and only minor differences in external traits, a situation that often leads to incorrect assignments [17]. DNA barcoding helps address this limitation by matching standardized loci to curated reference sequences, allowing taxa to be separated even when morphology provides little diagnostic resolution [18,19]. In fishes, the mitochondrial *COI* gene is one of the most widely used markers for this purpose because it is easily amplified and contains sufficient variation for species-level discrimination [20,21]. Evidence for its utility is accumulating across different regions [22]. For example, Linh et al. used *COI* gene barcoding to resolve 26 species of Gobiidae, complementing morphology-based identification and supporting fisheries conservation initiatives in Vietnam [23]. Xu et al. employed barcoding in the Dongsha Islands to identify fish species and improve inventories of local diversity [24]. Previous studies on *Schizothorax*, Schizothoracinae, and freshwater cyprinids have also shown that *COI* gene barcoding can support species identification, diagnostic-site screening, and regional barcode-library construction. Recent large-scale fish barcode libraries have further emphasized that voucher-based *COI* datasets remain valuable for integrated taxonomy, metabarcoding, eDNA surveys, biodiversity monitoring, and the detection of discordance between molecular operational taxonomic units and morphology-based identifications [25,26]. The Tarim River Basin provides a particularly relevant and challenging system for testing these issues. As an arid inland river basin, it contains fragmented tributaries, strong environmental gradients, and isolated aquatic habitats, all of which may restrict dispersal and promote population divergence. At the same time, historical hydrological connections, secondary contact among tributaries, and human-mediated habitat alteration may facilitate gene flow or obscure lineage boundaries. This combination of geographic isolation and potential admixture makes the basin a problematic context for species delimitation. Such systems are increasingly treated as taxonomically complex cases in which morphology, mitochondrial markers, geographic context, and, where possible, genome-wide evidence should be evaluated together rather than relying on a single line of evidence [15,27]. In addition, previous work has shown that endangered Schizothoracine fishes in the Tarim River Basin may be threatened by introgressive hybridization, especially between *A. laticeps* and *S. biddulphi* [12]. Thus, the Tarim River Basin is not only a conservation priority but also an informative natural system for evaluating whether morphology-based taxonomy is consistent with mitochondrial genetic structure.

Therefore, the present study combined diagnostic morphological characters with mitochondrial *COI* DNA barcoding to evaluate species identification and genetic differentiation among six Schizothoracine fishes from the Tarim River Basin, Xinjiang, China. Our central hypothesis was that morphologically recognized species would also display genetic divergence in *COI* gene sequences—manifested as species-specific haplotypes, clear interspecific genetic distances, and well-supported mitochondrial lineages [12,15,16]. We further hypothesized that, among closely related *Schizothorax* species in the same drainage system, incomplete lineage sorting, recent divergence, or historical gene flow could result in shared haplotypes and weak taxonomic resolution. Specifically, we aimed to test whether *COI* gene barcoding could (i) support species identification based on diagnostic morphology and mitochondrial sequences; (ii) assess barcode gap patterns and genetic-distance differences among the six taxa; (iii) detect haplotype sharing among closely related *Schizothorax* species; and (iv) clarify mitochondrial phylogenetic relationships among the sampled Schizothoracine fishes.

## 2. Materials and Methods

### 2.1. Experimental Materials

**Figure 1:**
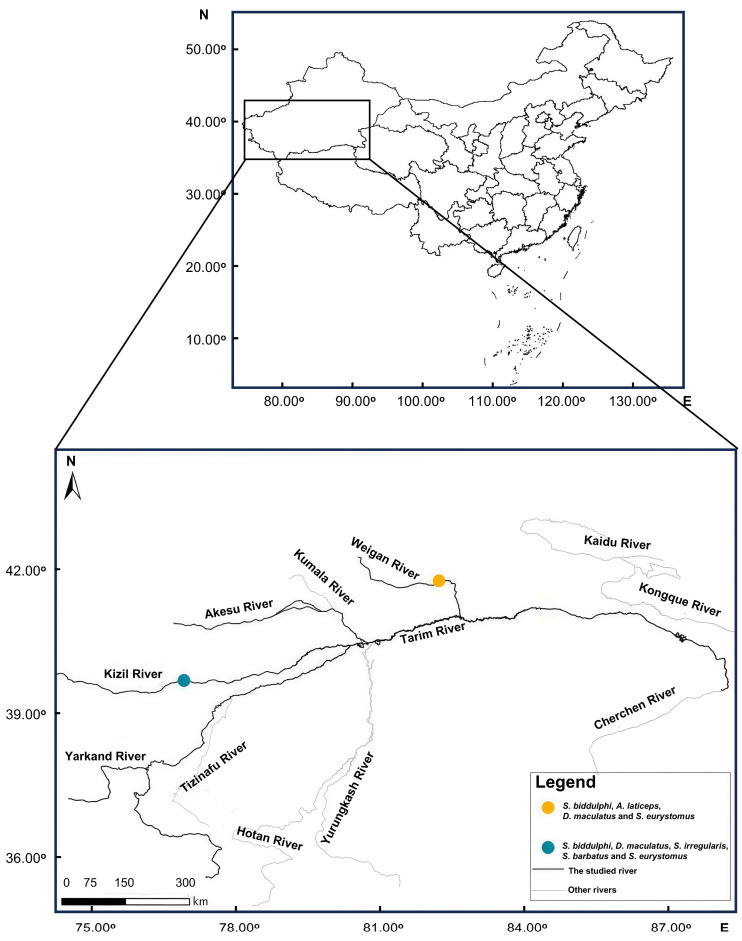
Schematic diagram of sampling point distribution. ○: sampling point (Yellow represents the Wei Gan River, and blue represents the Kizil River).

A total of 124 specimens from six Schizothoracine fish species were collected from the Kizil River and Weigan River in the Tarim River Basin using field net sampling. Fish were captured using gill nets and hand nets, depending on local habitat conditions. After collection, live specimens were temporarily maintained in aerated containers and transported to the sampling station for morphological examination and tissue sampling. The species collected were *S. biddulphi*, *A. laticeps*, *D. maculatus*, *S. irregularis*, *S. barbatus*, and *S. eurystomus* (Figure 1 and Table 1). Morphological identification was carried out according to the guidelines outlined in “Fishes of Xinjiang” [18]. Live specimens were anesthetized with MS-222 (tricaine methanesulfonate; Sigma-Aldrich, St. Louis, MO, USA) at 40 mg/L, and tail-fin tissue samples were placed in centrifuge tubes (Axygen, Union City, CA, USA) containing anhydrous ethanol (Sinopharm Chemical Reagent Co., Ltd., Shanghai, China). They were stored at −20 °C for future molecular identification. Specimen collection was conducted with permission from the local fishery administration authorities, and all experimental procedures were approved by the Animal Research Ethics Committee of Tarim University, China (approval no. PA20260310012).

### 2.2. DNA Extraction, Amplification, and Sequencing

Genomic DNA was extracted from approximately 50 mg of tail-fin tissue using an animal genomic DNA magnetic bead extraction kit provided by Tsingke Biotechnology Co., Ltd. (Beijing, China). Briefly, tissue samples were homogenized in 200 μL of PBS buffer and digested with Buffer gA1 and proteinase K supplied with the kit at 65 °C for 1–2 h. After centrifugation at 12,000× *g* for 5 min, the supernatant was transferred to a new 96-well plate. DNA was bound to magnetic beads in the presence of anhydrous ethanol, washed with Buffer PW and wash buffer, and eluted in 50 μL of sterile water. DNA integrity was checked using a 1.0% agarose gel (Biowest, Nuaillé, France), and PCR products were examined using a 1.5% agarose gel (Biowest, Nuaillé, France). The *COI* fragment was amplified using primers. The expected amplicon size was 656 bp. PCR was performed in a 40 μL reaction volume containing 20 μL of T8 High-Fidelity Master Mix (Tsingke Biotechnology Co., Ltd., Beijing, China), 2 μL of each primer, 2 μL of DNA template, and 14 μL of water. The PCR program was as follows: initial denaturation at 98 °C for 2 min; 35 cycles of 98 °C for 10 s, 58 °C for 10 s, and 72 °C for 20 s; followed by final extension at 72 °C for 5 min. PCR products were checked by agarose gel electrophoresis, and products with clear target bands were selected for Sanger sequencing by Tsingke Biotechnology Co., Ltd. (Beijing, China). Raw chromatograms were analyzed using Sequencing Analysis 5.2 (Applied Biosystems, Thermo Fisher Scientific, Waltham, MA, USA). Forward and reverse sequences were assembled using ContigExpress module in Vector NTI Advance v11.5 (Invitrogen/Thermo Fisher Scientific, Waltham, MA, USA). Low-quality terminal regions with a Phred quality score below Q20 and ambiguous bases with unclear or overlapping peaks were removed manually.

For the amplification of the *COI* gene, the primers used were: F (5′-TGGTGCCTGAGCCGGAATAG-3′) and R (5′-GGTGGCCAAAGAATCAGAATAAGTG-3′), which were synthesized by Tsingke Biotechnology Co., Ltd. (Beijing, China). Prepare a total PCR reaction volume of 40 μL by combining 20 μL of 2× T8 High-Fidelity Master Mix, 2 μL each of 10 μM forward primer (Primer F) and reverse primer (Primer R), 2 μL of DNA template, and supplementing the remainder with ddH_2_O to reach 40 μL. Set the amplification conditions as follows: pre-denaturation at 98 °C for 2 min in the first cycle, denaturation at 98 °C for 10 s, annealing at 58 °C for 10 s, and extension at 72 °C for 20 s in the subsequent 35 cycles; finally, extend at 72 °C for 5 min and store at 4 °C. Then, perform agarose gel electrophoresis of the PCR products by combining 2 μL of the sample with 6 μL of bromophenol blue and running at 300 V for 12 min. Extract DNA from electrophoresis patterns using a gel extraction kit and conduct sequencing using the Sanger method. After centrifuging the BDT reaction, add 38 μL of vortexed Ferrite Beads to the reaction plate and purify by washing with Magical Buffer. Choose a distinct band from the PCR product and send it to Tsingke Biotechnology Co., Ltd. (Beijing, China) for sequencing analysis.

### 2.3. Data Analysis

Raw *COI* gene chromatograms were checked and edited using Sequencing Analysis 5.2 (Applied Biosystems, Thermo Fisher Scientific, Waltham, MA, USA), the ContigExpress module in Vector NTI Advance v11.5 (Invitrogen/Thermo Fisher Scientific, Waltham, MA, USA), and Geneious Prime v2021.2.2 (Biomatters Ltd., Auckland, New Zealand). Low-quality ends and ambiguous bases were manually removed, and forward and reverse reads were assembled into consensus sequences. After manual trimming and assembly, the consensus *COI* sequences were aligned using Clustal W algorithm implemented in MEGA v11.0. Raw chromatograms were inspected to remove low-quality terminal regions and ambiguous base calls. Because *COI* is a mitochondrial protein-coding gene, the aligned sequences were translated into amino acids using the vertebrate mitochondrial genetic code to screen for stop codons, insertions, deletions, and frameshifts. Sequences showing such abnormalities were considered potential pseudogenes or nuclear mitochondrial sequence candidates and would be excluded from subsequent analyses. No stop codons, insertions, deletions, or frameshifts were detected in the final dataset. Sequence length, variable sites, conserved sites, parsimony-informative sites, and base composition of the *COI* gene were analyzed using MEGA v11.0 and DNAMAN v10.0 (Lynnon Biosoft, San Ramon, CA, USA). The genetic diversity index and the number of haplotypes were analyzed using DNASP v6.12.03. Pairwise genetic distances within and among species were estimated using simple uncorrected *p*-distances to evaluate genetic divergence and the presence of a DNA barcoding gap. A haplotype network was constructed using PopART v1.7 to visualize haplotype relationships and haplotype sharing among species. Species-delimitation analyses were conducted using complementary distance-based and tree-based approaches. ABGD was performed using the aligned 656-bp *COI* dataset and the uncorrected *p*-distance matrix through the ABGD web interface. The prior maximum intraspecific divergence value (*P*) was set from 0.001 to 0.10, with 10 recursive steps and a relative gap width (X) of 1.5. Both initial and recursive partitions were examined, and the stable partition was retained as the ABGD delimitation scheme [28]. The GMYC analysis was conducted using an ultrametric *COI* tree. The ultrametric tree was generated using BEAST v1.10.4 under the same nucleotide substitution model used for phylogenetic reconstruction, with two independent MCMC runs. Convergence was assessed using Tracer v1.7.2 by checking that effective sample size values were greater than 200, and the first 10% of sampled trees were discarded as burn-in. A maximum clade credibility tree was then using TreeAnnotator v1.10.4 and used for the single-threshold GMYC analysis implemented in the R package splits v1.0-20 [29]. The bPTP analyses were performed using the online bPTP server based on both the maximum-likelihood (ML) and Bayesian-inference (BI) *COI* topologies. Each analysis was run for 5.0 × 10^5^ MCMC generations, with a thinning interval of 100 and a burn-in of 10%. The resulting partitions were compared with the morphology-based species assignments and with the ABGD and GMYC outputs. Because all delimitation analyses were based on a single mitochondrial locus, the inferred partitions were interpreted as *COI*-based molecular operational taxonomic units rather than definitive species boundaries [30]. Data visualization was performed using R 4.6.0 and the ggplot2 package 4.0.3.

## 3. Results

### 3.1. Morphological Classification and Identification of Six Schizothoracine Fishes in Xinjiang

**Figure 2:**
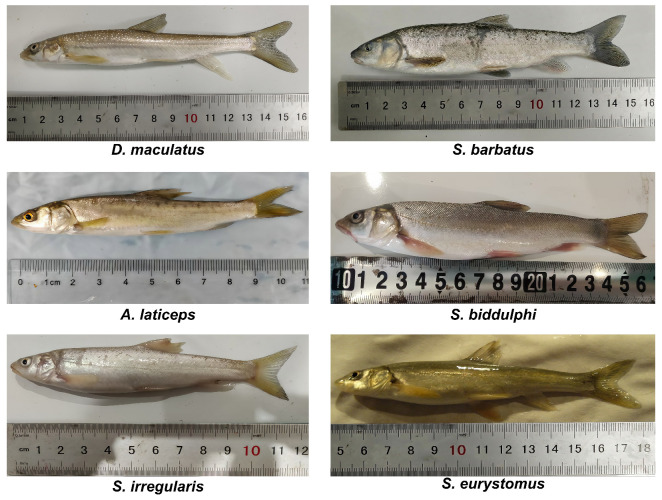
Six species of Schizothoracine fishes in Xinjiang.

A total of 124 cyprinid specimens were identified as belonging to three genera and six species based on the diagnostic keys and species descriptions provided in Fishes of Xinjiang [31] (Figure 2, Table 2). Among the specialized taxa, *D. maculatus* (*Diptychus*) is morphologically distinct, exhibiting a single pair of barbels, a naked thoracic-abdominal region, and two rows of pharyngeal teeth (3·4/4·3). *Aspiorhynchus laticeps* has one pair of barbels, a large head, a lower jaw longer than the upper jaw, and a flattened snout. It also has pharyngeal teeth arranged in three rows (2·3·5/5·3·2). In contrast, the *Schizothorax*—represented here by *S. biddulphi*, *S. barbatus*, *S. irregularis*, and *S. eurystomus*—uniformly displays two pairs of barbels and three rows of pharyngeal teeth, yet diverges in lip structure and keratinization. *Schizothorax barbatus* features two pairs of barbels, an elongated body, a blunt conical head, a longer maxilla than mandible, a protruding upper lip, an enlarged lower lip with a free posterior margin, and pharyngeal teeth in three rows (2·3·5/5·3·2), among other characteristics. *Schizothorax irregularis* has two pairs of barbels, an elongated body, a conical head, a blunt snout, a longer upper jaw than lower jaw, thick lips, an enlarged lower lip often separated by a lip flap, and pharyngeal teeth in three rows (2·3·5/5·3·2), among other traits. For instance, *S. eurystomus* is differentiated by the presence of sharp, transverse keratinous projections on the lower jaw and a widely interrupted lower lip, traits absent in the morphologically similar *S. biddulphi*.

### 3.2. Analysis of COI Gene Sequence Fragments

**Figure 3:**
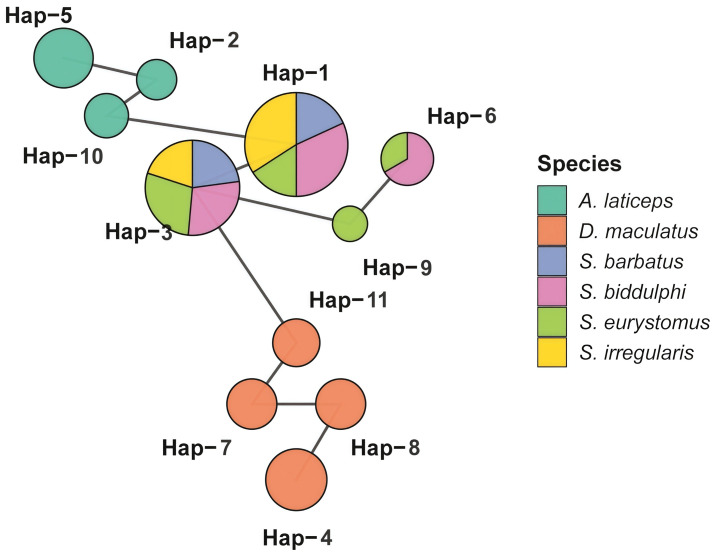
Haplotype relationship diagram of *COI* sequences from six Schizothoracine fishes from Xinjiang. Circle size represents haplotype frequency, and colours indicate species.

Bidirectional sequencing of the *COI* gene was performed for 124 individuals from six Schizothoracine fishes in Xinjiang. This analysis produced a 656 bp consensus sequence with no insertions or deletions. Within the *COI* gene, the sequence contained 106 variable sites (16.30% of the total bases) and 549 conserved sites (83.70% of the total bases). A total of 104 parsimony-informative sites (Pi) were identified, which accounted for 15.93% of the total bases. These results indicate that the *COI* gene fragment contained sufficient variation for subsequent haplotype and genetic-diversity analyses. The (A + T)% content exceeded the (G + C)% content in all six species, suggesting a significant AT bias, consistent with the typical characteristics of the *COI* gene in teleost fish [32]. As shown in Table 3, G was the lowest-frequency base in all six taxa. Genetic diversity analysis showed that nucleotide diversity indices (π) ranged from 0.00073 to 0.0027, and haplotype diversity indices (Hd) ranged from 0.44 to 0.75 among the six *Schizothorax* species. *Diptychus maculatus* showed the highest observed genetic diversity indices in this dataset (Hd = 0.75, π = 0.0027), suggesting relatively higher mitochondrial variation among the sampled individuals. By contrast, *S. irregularis* showed the lowest genetic diversity, with Hd = 0.44 and π = 0.00073. Using DNASP 6.0, 11 haplotypes were identified. Hap-1 was the most common haplotype, with 44 occurrences, followed by Hap-3 with 35 occurrences. Importantly, Hap-1 and Hap-3 were shared among all four *Schizothorax* species, namely *S. biddulphi*, *S. eurystomus*, *S. irregularis*, and *S. barbatus*, indicating extensive haplotype sharing and limited *COI* gene differentiation within this group. In contrast, Hap-5 was the predominant haplotype in *A. laticeps*, while Hap-4 dominated in *D. maculatus* (Table 3). The haplotype relationship diagram showed that Hap-1 and Hap-3 were shared central haplotypes among the four *Schizothorax* species, whereas the haplotypes of *D. maculatus* and *A. laticeps* were more distinct (Figure 3). Upon conducting BLAST v2.17.0 searches of this fragment in GenBank, it was found that both the Hap-1 and Hap-3 haplotypes displayed match rates of over 97% within the *Schizothorax*. Haplotype Hap-4 exhibited a 98.04% similarity with *D. maculatus* (MN_413609.1), while Hap-5 showed a 99.92% similarity with *A. laticeps* (KY_436760.1). The species have been confirmed as *D. maculatus* and *A. laticeps*, respectively. These findings, in conjunction with morphological characteristics, provide further support for their accurate species identification. In contrast, the shared Hap-1 and Hap-3 among several *Schizothorax* species indicate limited *COI* gene resolution within this group.

### 3.3. Genetic Distance Analysis of Six Schizothorax Species in Xinjiang

Based on the *COI* sequence variation and haplotype patterns described above, we further estimated genetic distances and conducted species-delimitation analyses to evaluate molecular divergence among the six Schizothoracine taxa. Analysis of intraspecific and interspecific genetic distances among six *Schizothorax* species in Xinjiang (Table 4) indicated that interspecific genetic distances varied from 0.262% to 15.296%. Strong divergence was observed among the major lineages/genera, especially between *D. maculatus* and the other taxa. The most notable interspecific genetic distance was found between *D. maculatus* and *S. barbatus* (15.296%). This was followed by the genetic distance between *D. maculatus* and *S. irregularis* (14.352%), both significantly surpassing Hebert’s proposed 2% threshold for species identification [33]. Intraspecific genetic distances within *Schizothorax* fishes were all below 0.5%, indicating relatively low sequence divergence within each species. In contrast, divergence within *Schizothorax* was weak. Among the four *Schizothorax* species, interspecific genetic distances were low, with the smallest value detected between *S. barbatus* and *S. irregularis* (0.262%). These values were close to or only slightly higher than their intraspecific distances, suggesting weak *COI* gene differentiation within the *Schizothorax* group. Therefore, the four *Schizothorax* species did not form a clear barcoding gap, in contrast to the much larger distances observed between *D. maculatus* and the other taxa.

**Figure 4:**
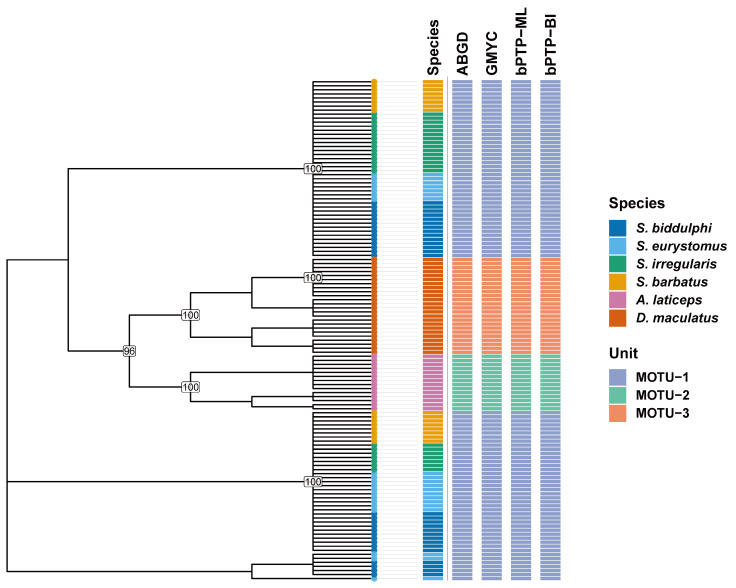
Maximum-likelihood phylogenetic tree of six Schizothoracine fishes based on *COI* sequences, with species-delimitation results shown as annotation tracks.

Based on the pairwise distance matrix, the ABGD analysis divided the six Schizothoracine fishes into three molecular units. The first unit included *S. biddulphi*, *S. eurystomus*, *S. irregularis*, and *S. barbatus*, whereas *D. maculatus* and *A. laticeps* each formed independent units. This grouping pattern indicated that *D. maculatus* and *A. laticeps* displayed clear barcode gaps and could be distinguished from the other taxa, while no clear barcode gap was detected among the four *Schizothorax* species. The GMYC and bPTP analyses, including both ML and BI implementations of bPTP, produced delimitation schemes consistent with the ABGD result. To make these results explicit, Figure 4 presents the species-delimitation results on the ML topology, with the nominal species assignments and the ABGD, GMYC, bPTP-ML, and bPTP-BI results shown as separate annotation tracks. These analyses consistently supported *D. maculatus* and *A. laticeps* as independent molecular lineages, whereas *S. biddulphi*, *S. eurystomus*, *S. irregularis*, and *S. barbatus* were not clearly delimited as separate *COI*-based molecular units.

Because *F*-statistics and *F_ST_*-derived *N_m_* estimates are primarily designed for population-level comparisons within a species or metapopulation framework, they were not used here to infer differentiation or gene flow among nominal species. Therefore, species-level interpretations in this study were based on *COI p*-distances, barcode-gap patterns, haplotype sharing, and the results of ABGD, GMYC, and bPTP analyses. These combined results consistently indicated strong mitochondrial separation of *D. maculatus* and *A. laticeps*, but limited *COI*-based resolution among *S. biddulphi*, *S. eurystomus*, *S. irregularis*, and *S. barbatus*.

### 3.4. COI Lineage Relationships Based on the ML Topology

The ML topology based on the complete 656-bp *COI* gene sequences provided a tree-based visualization of mitochondrial lineage relationships among the six Schizothoracine fishes (Figure 4). In this topology, *Diptychus maculatus* formed a clearly separated and deeply divergent *COI* lineage, and *A. laticeps* was also recovered as an independent mitochondrial lineage. These results were consistent with their distinct haplotypes, relatively large *COI p*-distances, clear barcode gaps, and the molecular units inferred by ABGD, GMYC, and bPTP. In contrast, the four nominal *Schizothorax* species, namely *S. biddulphi*, *S. eurystomus*, *S. irregularis*, and *S. barbatus*, were not resolved as species-specific *COI* lineages. Individuals assigned to these taxa were placed within a poorly resolved *Schizothorax* assemblage with short internal branches, consistent with the shared Hap-1 and Hap-3 haplotypes and the low interspecific *COI* distances observed among them. The annotation tracks in Figure 4 further showed that ABGD, GMYC, bPTP-ML, and bPTP-BI assigned these four nominal species to the same molecular unit, whereas *D. maculatus* and *A. laticeps* were each delimited as separate units. Overall, the ML topology and species-delimitation results supported the distinct mitochondrial lineages of *D. maculatus* and *A. laticeps*, but indicated limited *COI*-based resolution among the closely related *Schizothorax* species.

## 4. Discussion

### 4.1. Genetic Diversity of the COI Gene in Six Schizothoracine Fishes

Environmental heterogeneity across the Qinghai–Tibet Plateau provides an important context for interpreting genetic variation in Schizothoracine fishes, and genetic diversity is often treated as a practical measure of their evolutionary potential. Recent genomic studies of Schizothoracine fishes also indicate that plateau river systems and high-altitude environments can shape population structure and adaptive signals related to energy metabolism, DNA repair, hypoxia response, and conservation vulnerability [34,35]. In this regard, haplotype diversity (Hd) and nucleotide diversity (π) offer complementary information on mitochondrial variation within each sampled nominal taxon and can help describe historical demography and persistence [36,37]. It should be noted that these indices describe within-taxon genetic diversity and were not used here as evidence of interspecific differentiation or species delimitation. Therefore, the comparisons in this section are descriptive and refer only to relative levels of *COI* diversity within the sampled taxa. All six taxa shared a pronounced AT bias in *COI* gene sequences, consistent with the nucleotide composition typical of teleost mitochondria and comparable to patterns reported in *Triplophysa* and Siluriformes [32,38,39]. The AT-rich pattern observed in all six taxa supports the mitochondrial origin and reliability of the amplified *COI* gene fragments. This compositional bias is also consistent with the sequence characteristics reported in many teleost mitochondrial *COI* gene datasets, indicating that the dataset was suitable for subsequent genetic-diversity and phylogenetic analyses. The levels of within-taxon mitochondrial diversity were not uniform among the sampled nominal taxa, possibly reflecting the combined influence of altitude-related environmental conditions, demographic history, and recent human disturbance. Elevational gradients have been associated with increased genetic variation, whereas intensive exploitation and habitat degradation can rapidly erode diversity via demographic bottlenecks, as seen in *Scatophagus argus* [40,41]. The taxa also occupy different altitudinal niches: *A. laticeps* occurs largely below 1500 m, *Schizopygopsis* species are concentrated between 1300 m and 2700 m, and *D. maculatus* is primarily distributed from 2700 m to 3700 m [42]. In line with its high-altitude distribution, *D. maculatus* showed the highest within-taxon mitochondrial diversity in the present dataset (Hd = 0.75, π = 0.0027), which may reflect long-term persistence and accumulation of haplotype variation under cold and hypoxic conditions [43]. This pattern was also reflected in the haplotype composition, as *D. maculatus* possessed four haplotypes that were not shared with the other taxa. By contrast, *S. irregularis* showed the lowest diversity and contained only two shared haplotypes, suggesting relatively limited mitochondrial variation in the sampled individuals. These results indicate that mitochondrial genetic diversity was unevenly distributed among the six sampled nominal taxa, but they should not be interpreted as direct evidence of species-level differentiation.

### 4.2. Comparison of Morphological Identification and COI Barcoding

Because several morphological traits used for *Schizothorax* identification may overlap among species, morphology alone is sometimes insufficient for reliable delimitation. To complement these observations, we conducted *COI*-based DNA barcoding analyses to assess genetic differentiation and phylogenetic relationships among the studied taxa. Morphological characters remain central to *Schizothorax* taxonomy, yet many traits used for diagnosis are not entirely stable and can shift with both genetic background and environmental plasticity [44]. Against this background, *D*. *maculatus* appears relatively specialized, showing sparse scalation and reduced barbels. Such characters may represent high-altitude adaptations in fast-flowing plateau rivers, where energetic constraints and feeding conditions differ markedly from lowland habitats. By comparison, *A*. *laticeps* and the *Schizothorax* species examined here are generally considered members of the primitive group, sharing complete body scalation and three rows of pharyngeal teeth. Barbel number provides one of the clearer distinctions within this assemblage: *A. laticeps* bears a single pair of barbels, whereas *Schizothorax* species typically possess two pairs. Ecological conditions may further shape external morphology, as fishes in lentic or slow-flowing waters often show trends toward omnivory or carnivory, accompanied by reduced barbels and a broader mouth, with the lower jaw extending beyond the upper jaw [45]. At finer taxonomic resolution, species discrimination within *Schizothorax* frequently depends on lip structure and the keratinized edge of the lower jaw [46]. In our material, *S. barbatus* and *S. irregularis* differed only subtly despite their generally thick lips: the former had a free posterior lower-lip margin, whereas the latter usually displayed a lip flap that separates the two sides. Likewise, *S. biddulphi* closely resembled *S. eurystomus*, but the keratinized lower-jaw edge in *S. eurystomus* disrupted the lower lip and produced two lobes separated by a distinct gap.

A key assumption of *COI* gene barcoding is the existence of a “barcoding gap”, where between-species divergence clearly exceeds within-species variation. Hebert et al. noted that *COI* gene distances are often >2% among species but <1% within species [20], and Bloom et al. suggested that a practical barcode typically requires interspecific divergence to exceed intraspecific divergence by at least ~10-fold [47]. ABGD further formalizes this concept by treating samples within the same partition as a single putative species [28]. However, the 2% *COI* gene distance threshold should be regarded as a commonly cited comparative guideline rather than a universal or absolute criterion for species delimitation, because mitochondrial divergence can vary among taxa and evolutionary contexts. Recent integrative-taxonomy studies similarly emphasize that barcode gaps and single-locus mitochondrial partitions should be interpreted cautiously in taxonomically complex groups, especially when recent divergence, introgression, incomplete lineage sorting, or geographic population structure may influence the observed genetic pattern [15,27]. Here, *COI* gene barcoding performed well for *A. laticeps* and *D. maculatus*: their interspecific divergence was 13.171%, approximately 48 times higher than intraspecific variation, and both ABGD, GMYC and bPTP consistently separated them into distinct groups, matching morphological expectations. This conclusion was supported by multiple lines of evidence, including their distinct dominant haplotypes, large interspecific distances, clear barcode gaps, and independent lineages in Figure 4. In contrast, the *Schizothorax* assemblage showed weaker resolution. The lowest interspecific distance was observed between *S. barbatus* and *S. irregularis*, and Hap-1 and Hap-3 were shared among four *Schizothorax* species. These results suggest that *COI* gene sequence variation was insufficient to fully separate these closely related taxa in the present dataset. The mismatch between morphological assignments and molecular partitions (e.g., “*S. irregularis*” specimens clustering with *S. biddulphi*) implies that some diagnostic characters may reflect ecological plasticity rather than reproductive isolation [48]. Comparable morphology–barcode discordance has been described in other *Schizothorax* taxa, including *S. progastus* and *S. richardsonii* and multiple species from the Yarlung Tsangpo River Basin [49,50]. Such patterns are consistent with habitat-associated morphological divergence occurring without parallel mitochondrial differentiation, leaving *COI* gene distances below the conventional ~2% criterion for species-level separation [33]. Haplotype sharing was considered evidence of genetic connectivity or incomplete lineage separation, not a direct criterion for species delimitation. Species delimitation was based on the complete *COI* gene alignment, whereas haplotype analysis was used only to visualize haplotype composition and sharing patterns. Therefore, the lack of clear *COI* gene separation should not, by itself, be taken as sufficient evidence to reject the morphologically recognized species. Instead, it suggests that a single mitochondrial marker has limited resolution for delimiting these closely related taxa. The observed pattern may reflect recent divergence, incomplete lineage sorting, introgression, or a combination of these processes, but these explanations remain putative in the absence of nuclear loci or genome-wide data. Future studies incorporating multilocus nuclear markers and broader sampling will be necessary to test these hypotheses more rigorously.

### 4.3. COI-Based Phylogenetic Relationships Among Six Schizothoracine Fishes

Mitochondrial barcodes may provide preliminary insight into phylogeographic patterns by linking lineage structure to possible drainage connectivity. However, such interpretation should be made cautiously because the present sampling was limited to the Kizil River and Weigan River within the Tarim River Basin. In river networks, taxa occurring in close geographic proximity may retain greater genetic affinity, but confirming this pattern requires broader geographic sampling and explicit evidence from drainage-history studies [51]. Building on the scenario proposed by Hai Sa et al., Schizothoracine fishes may have originated in the Western Kunlun Mountains, with early lineages becoming isolated between the Northern Tianshan and Western Kunlun ranges [52]. Subsequent uplift within the Tarim Basin may have altered dispersal corridors, allowing secondary contact in some areas while isolating others, particularly following the uplift of the Southern Tianshan Mountains. Within this context, *D. maculatus* showed the strongest mitochondrial differentiation from the other taxa, as indicated by its large *COI p*-distances, distinct haplotypes, clear barcode gap, and separate position in the *COI* clustering analysis. This result was also consistent with the species-delimitation analyses, which recovered *D. maculatus* as an independent *COI*-based molecular unit. In contrast, *A. laticeps* was genetically closer to the *Schizothorax* species than *D. maculatus* was, but it was still recovered as a separate *COI*-based molecular lineage by ABGD, GMYC, and bPTP. However, the present *COI* data alone are insufficient to evaluate its genus-level taxonomic placement or broader relationship with *Schizothorax*. Meanwhile, the four Tarim Basin *Schizothorax* species showed minimal *COI* divergence and shared Hap-1 and Hap-3, indicating weak mitochondrial separation within this closely related group. Together with the shared Hap-1 and Hap-3 haplotypes, this pattern may explain why the four species were not resolved as independent *COI* gene lineages in the *COI* clustering analysis. Such patterns may be consistent with recent divergence, incomplete lineage sorting, introgressive hybridization in sympatry, or a combination of these processes [53,54], while ecotypic differentiation may still promote phenotypic diversification without complete mitochondrial differentiation [55]. Our *COI* results indicate relatively weak mitochondrial differentiation among the four *Schizothorax* species, but stronger mitochondrial divergence between *Schizothorax* and the taxa assigned to other morphologically recognized genera, especially *D. maculatus* and *A. laticeps*. This pattern suggests that geographic isolation and historical drainage connectivity may have contributed to the observed mitochondrial structure, although the *COI*-only data do not allow us to determine whether geography is the primary driver of genetic differentiation. Overall, the phylogenetic pattern supports a hierarchical interpretation of the *COI* results: *COI* gene barcoding is effective for distinguishing deeply divergent lineages such as *D. maculatus* and *A. laticeps*, but has limited discriminatory power among closely related *Schizothorax* species. Therefore, the phylogeographic explanation proposed here should be regarded as a preliminary hypothesis rather than a definitive reconstruction of drainage history, and should be tested in future studies using wider sampling across tributaries, historical drainage data, and nuclear or genome-wide markers. This interpretation is consistent with recent genomic species-delimitation studies showing that sampling design, geographic structure, and genome-wide evidence can substantially influence the validation of species hypotheses, particularly when mitochondrial markers alone provide limited resolution [27]. Similarly, *COI* alone cannot resolve the subfamily-level placement of *Diptychus* or its relationship to Schizothoracinae, and this issue will require broader taxon sampling and multilocus phylogenetic evidence.

## 5. Conclusions

In this study, *COI* gene barcoding was used to evaluate species identification and genetic relationships among six Schizothoracine fishes from Xinjiang. The results showed that *COI* sequences contained sufficient variation to distinguish major mitochondrial lineages. *Diptychus maculatus* and *Aspiorhynchus laticeps*, which are assigned to different genera from *Schizothorax* based on diagnostic morphology, were recovered as distinct mitochondrial lineages, consistent with their morphology-based taxonomic assignments and species-delimitation results. In contrast, the four *Schizothorax* species, including *S. biddulphi*, *S. eurystomus*, *S. irregularis*, and *S. barbatus*, showed low genetic divergence, shared Hap-1 and Hap-3 haplotypes, and did not form well-supported species-specific clades. These findings indicate that *COI* gene barcoding is useful for identifying distinct morphology-based genera and major mitochondrial lineages, but has limited resolution for closely related *Schizothorax* species within the Tarim River Basin, possibly due to recent divergence, incomplete lineage sorting, or historical gene flow. Therefore, the present *COI*-only evidence should be interpreted as complementary to, rather than a replacement for, morphology-based generic classification. Any future reassessment of relationships among *Aspiorhynchus*, *Diptychus*, and *Schizothorax* will require stronger evidence from multilocus nuclear markers, genome-wide data, broader taxon sampling, and integrated morphological analyses. From a conservation perspective, expanding the regional *COI* gene barcode reference library will improve species authentication, biodiversity surveys, and long-term monitoring of native Schizothoracine fishes in Xinjiang, particularly for conservation-relevant lineages such as *D. maculatus* and *A. laticeps*. Future genomic follow-up will be essential for clarifying species boundaries, detecting possible introgression, and defining appropriate conservation units.

## Figures and Tables

**Table 1:** Sampling information for six Schizothoracine fishes from the Tarim River Basin.

Locality	Latitude and Longitude	Species	Numbers
Kizil River	39.55 N, 76.54 E	*D. maculatus*	15
		*S. biddulphi*	10
		*S. eurystomus*	3
		*S. barbatus*	16
		*S. irregularis*	22
Weigan River	41.46 N, 82.30 E	*D. maculatus*	9
		*A. laticeps*	14
		*S. biddulphi*	18
		*S. eurystomus*	17

**Table 2:** Basic information of samples of six *Schizothorax* fishes.

Genus	Species	Count	Body Length (mm)	Body Weight (g)	Characteristics
*Diptychus*	*D. maculatus*	24	85.00~100.78	7.43~11.91	One pair of antennae, mouthparts positioned low, mandibles with sharp, horny tips, and irregular spots.
*Aspiorhynchus*	*A. laticeps*	14	57.63~65.76	10.23~12.22	One pair of antennae, mouthparts terminal, mouth slit wide, snout flattened.
*Schizothorax*	*S. biddulphi*	28	62.75~82.28	4.42~7.62	Two pairs of antennae, mouthparts in a low position, lower lip narrow, rostrum pointed, and mandibles lacking a keratinous margin.
	*S. eurystomus*	20	184.47~195.34	75.73~83.27	Two pairs of antennae, mouthparts in a lower position, a small mouth opening, and mandibles with sharp keratinized tips.
	*S. barbatus*	16	107.24~123.21	27.33~33.21	Two pairs of antennae, mouthparts in a lower position, lower lip enlarged and with two fused lobes.
	*S. irregularis*	22	105.76~108.66	14.58~16.53	Two pairs of antennae, lower lip enlarged and discontinuous between the two lobes, mandibles lacking a keratinized margin.

**Table 3:** Base composition of the *COI* gene and genetic diversity parameters of six *Schizothorax* fishes.

Species	Base Content (%)				Number of Haplotypes	Haplotype Composition	Haplotype Diversity Index (Hd)	Nucleotide Diversity Index (π)
	A	T	C	G				
*S. biddulphi*	25.6	27.5	29.1	17.8	3	Hap-1 (14 times)	0.59	0.0020
						Hap-3 (10 times)		
						Hap-6 (4 times)		
*D*. *maculatus*	27.2	27.9	27.6	17.3	4	Hap-4 (10 times)	0.75	0.0027
						Hap-7 (5 times)		
						Hap-8 (5 times)		
						Hap-11 (4 times)		
*S. irregularis*	25.6	27.7	29.1	17.7	2	Hap-1 (15 times)	0.44	0.00073
						Hap-3 (7 times)		
*S. eurystomus*	29.7	24.9	27.5	17.9	4	Hap-1 (7 times)	0.69	0.0023
						Hap-3 (10 times)		
						Hap-6 (2 times)		
						Hap-9 (1 time)		
*S. barbatus*	26.4	27.3	28.9	17.4	2	Hap-1 (8 times)	0.50	0.0015
						Hap-3 (8 times)		
*A. laticeps*	26.5	27.6	28.9	17.1	3	Hap-2 (2 times)	0.52	0.0021
						Hap-5 (9 times)		
						Hap-10 (3 times)		

**Table 4:** The intraspecific and interspecific uncorrected *p*-distances (%) of six Schizothoracine fishes in Xinjiang.

Species	*S. biddulphi*	*A. laticeps*	*D. maculatus*	*S. irregularis*	*S. barbatus*	*S. eurystomus*
*S. biddulphi*	**0.132**					
*A. laticeps*	3.215	**0.185**				
*D. maculatus*	12.081	13.171	**0.273**			
*S. irregularis*	0.429	5.087	14.352	**0.212**		
*S. barbatus*	0.463	5.104	15.296	0.262	**0.239**	
*S. eurystomus*	0.275	4.338	13.224	0.411	0.397	**0.162**

Note: The diagonal (bolded) represents intraspecific genetic distances; below the diagonal are interspecific genetic distances.

## Data Availability

The raw data supporting the conclusions of this article will be made available by the authors on request.

## Abbreviations

The following abbreviations are used in this manuscript:

*A. laticeps*	*Aspiorhynchus laticeps* (Day, 1877)
*D. maculatus*	*Diptychus maculatus* (Steindachner, 1866)
*S. barbatus*	*Schizothorax barbatus* (McClelland & Griffith, 1842)
*S. biddulphi*	*Schizothorax biddulphi* (Günther, 1876)
*S. eurystomus*	*Schizothorax eurystomus* (Kessler, 1872)
*S. irregularis*	*Schizothorax irregularis* (Day, 1877)
